# School‐Age Differences in Reasonable Accommodations for Students Who Stutter: A Content Analysis of Medical Letters

**DOI:** 10.1111/ped.70566

**Published:** 2026-10-29

**Authors:** Yoshikazu Kikuchi, Yumi Yamaguchi, Masamutsu Kenjo, Eriko Yoshida, Aoi Sato, Takashi Nakagawa

**Affiliations:** ^1^ Department of Otorhinolaryngology, Graduate School of Medical Sciences Kyushu University Fukuoka Japan; ^2^ Department of Special Needs Education University of Teacher Education Fukuoka Fukuoka Japan; ^3^ Faculty of Nursing and Nutrition University of Nagasaki Nagasaki Japan

**Keywords:** accommodations, peer interaction, school children, social anxiety, stuttering

## Abstract

**Background:**

Japanese schools must provide reasonable accommodations for students who stutter, but developmental variations in these needs are poorly understood. This study characterized accommodation requests in clinician‐drafted medical letters to clarify these needs.

**Methods:**

Medical letters for 116 students who stutter (65 elementary, 51 secondary) were retrospectively analyzed. Accommodation content was classified using inductive content analysis. Group proportions were compared, and stuttering frequency (%SS) and social anxiety (LSAS‐CA) were analyzed descriptively.

**Results:**

Five accommodation categories were identified. Elementary students significantly more frequently requested teacher‐mediated protective support, such as reporting teasing and classroom stuttering explanations. Secondary students more frequently requested social functioning accommodations, including subject‐teacher information sharing and entrance examination support. Presentation‐related accommodations were highly prevalent across both groups (61.2% overall). Participants who completed the anxiety assessment exhibited elevated social anxiety (mean LSAS‐CA: 42.1).

**Conclusion:**

Accommodation needs reflect structural school transitions, shifting from classroom protection under the elementary homeroom system to social participation support under the secondary subject‐teacher system. Importantly, presentation‐related needs represent a consistent concern, likely driven by subjective anxiety rather than objective stuttering severity. Medical letters may serve as valuable tools for initiating constructive clinician–family–school dialogue to facilitate tailored support.

## 1 Introduction

### 1.1 Epidemiology and Clinical Characteristics of Stuttering

Stuttering is a neurodevelopmental disorder characterized by disruptions in speech fluency that typically begin in early childhood [1, 2, 3]. Although approximately 74% of children recover naturally within 4 years of onset [4], stuttering that persists clearly beyond age eight is likely to continue into adolescence [5]. Although stuttering is fundamentally a disorder of speech timing [6], it exhibits distinctive clinical characteristics, such as the “choral speech effect,” in which symptoms diminish during unison reading [7, 8, 9, 10, 11]. Dopaminergic dysfunction is believed to play a central role in the etiology of stuttering [6, 12]. Although several pharmacological agents have shown efficacy in randomized controlled trials involving adults who stutter [13, 14], none have been approved by the US Food and Drug Administration [15]. Furthermore, although speech therapy provided by speech–language pathologists often improves fluency in clinical settings, many individuals struggle to generalize and maintain fluent speech in everyday contexts, including school environments [16].

### 1.2 Covert Stuttering and Secondary Social Anxiety Disorder

Students often employ “technologies of communication,” such as filler words (e.g., “uh,” “um,” “like”) or word substitution, to reduce social penalties. As a result of these sustained efforts, students may appear fluent, a phenomenon described as covert stuttering or “passing as fluent” [17]. However, overt stuttering severity does not necessarily correlate with subjective burden; therefore, students who appear “mild” due to covert stuttering may nonetheless experience substantial internal impairment [18]. Accordingly, assessment should address not only observable speech symptoms but also the presence of social anxiety disorder (SAD). SAD typically has an early onset, with approximately 50% of cases emerging by Age 11 and 80% by Age 20 [19]. These findings indicate that junior and senior high school years represent a critical developmental period for anxiety onset. Indeed, approximately 50% of adolescents who stutter meet diagnostic criteria for SAD, a comorbidity that substantially increases the risk of school refusal [20].

### 1.3 Multifaceted Barriers in School Life: Academic Participation and Peer Relationships

In academic settings, students who stutter face systemic barriers that limit meaningful participation. Teachers often lack adequate understanding of stuttering and may require students to read aloud despite requests for exemption, thereby undermining their ability to engage fully in learning [21]. Furthermore, the cognitive burden associated with concealing stuttering—such as mentally tracking upcoming reading passages—often distracts students from instruction, leading to missed learning opportunities [22]. These cumulative barriers may result in lower educational attainment [23] or severe withdrawal; notably, 26% of adolescents who stutter were classified in a “school refusal group,” often driven by comorbid SAD [20].

With respect to peer relationships, children who stutter face a heightened risk of teasing and bullying. Kikuchi et al. reported that 66.6% of children who stutter experienced negative peer behaviors, including imitation or ridicule [24]. Such victimization contributes to social isolation, as bystanders often fail to intervene because of limited awareness [25]. Moreover, exclusion from group activities deprives these students of opportunities to develop essential teamwork skills [21]. Although students often express a desire for peers to “treat them naturally” [26], fear of negative evaluation frequently enforces a “code of silence,” preventing help‐seeking behaviors [27].

### 1.4 Legal Framework for Educational Equity in Japan

Japan has aligned its policies with the global shift toward the social model of disability to address these barriers [28]. A central element of this is the Act for Eliminating Discrimination against Persons with Disabilities, enforced in 2016, which mandates the provision of reasonable accommodations in public schools [29]. To understand the accommodation needs within this framework, the structural characteristics of the Japanese school system must be considered. In Japan, elementary education (Grades 1–6) operates under a “homeroom teacher system,” where a single teacher manages most subjects and classroom life, fostering a close, protective environment. In contrast, secondary education (Grades 7–12) shifts to a “subject teacher system,” where students interact with multiple teachers daily. Furthermore, secondary education is heavily influenced by high‐stakes high school and university entrance examinations, which often include interviews. Despite the legal mandate, educators often lack sufficient knowledge regarding stuttering. Consequently, the educational system must prioritize equity—defined as fairness—rather than equality, which entails treating all students identically.

### 1.5 Purpose of This Study

In principle, classroom equity should be established through dialogue between students and teachers. However, students often struggle with self‐advocacy due to developmental immaturity or anxiety, and teachers may not fully grasp the invisible burdens of stuttering. To bridge this gap, specialist physicians play a crucial role by drafting medical letters. These letters are not merely diagnostic certificates but serve as advocacy tools derived from “constructive dialogue” between the specialist, the child, and their guardians. Regarding student needs, previous studies have identified subjective accommodation requests using questionnaires [26]; however, these relied on free‐text responses and did not capture accommodations formulated through clinical dialogue. Furthermore, there is a lack of quantitative data on what specific accommodations are deemed medically and educationally necessary by specialists to ensure equity. This study aims to characterize the reasonable accommodations requested for students who stutter in Japanese school settings through a content analysis of clinician‐drafted medical letters. By analyzing these documents, we seek to clarify the distinct support needs across developmental stages (elementary vs. secondary) as a form of needs assessment, serving as a foundation for effective school collaboration.

## 2 Methods

### 2.1 Participants

Of the students who presented to Kyushu University Hospital for stuttering evaluation and treatment between April 2016 and December 2022, this cross‐sectional study included 116 elementary and secondary school students (mean age: 11.7 years; 86 boys, 30 girls) for whom a medical letter requesting reasonable accommodations was drafted. The sample comprised 65 elementary school students and 51 secondary school students (junior high and high school). Students who attended a stuttering consultation during the study period but for whom no medical letter was drafted (due to lack of request or need) were excluded.

### 2.2 Data Source: Medical Letters for Reasonable Accommodation

The analyzed data were derived from medical letters requesting reasonable accommodations (hereafter referred to as “the letters”). These documents serve as formal requests from the medical institution to the school, advocating for environmental adjustments under the Act for Eliminating Discrimination against Persons with Disabilities. All letters were drafted by a single physician (the first author), a specialist with over 15 years of clinical experience in stuttering. Crucially, the content of these letters was not unilaterally determined by the physician. Instead, it was formulated through a shared decision‐making process involving the student, their guardians, and the physician.

### 2.3 Procedures

#### 2.3.1 Clinical Interview and Needs Assessment

Each letter was drafted following an approximately one‐hour comprehensive clinical interview. During this session, the physician assessed the student's speech characteristics and systematically explored specific difficulties in school life. The assessment covered:

- Speaking situations: Reading aloud in class, presentations, and day duty tasks (e.g., *Nichoku*, which involves leading class greetings or meetings).
- Peer interactions: Experiences of teasing, bullying, or exclusion
- Psychological state: Anxiety regarding speaking situations and school attendance. Based on this assessment, the physician proposed specific accommodations. The final content of the letter was determined only after obtaining consensus from the student and guardians that the requests accurately reflected their needs and preferences. Specifically, a draft review process was implemented. The physician presented a draft of the letter to the student and guardians and provided time for them to review the content. Revisions were made immediately if the phrasing did not fully capture their specific nuances or intent. The final version was issued only after confirming that the document accurately represented their voice.
- To assess the objective severity of stuttering, stuttering frequency (percentage of syllables stuttered, %SS) was evaluated using the Japanese Standardized Test for Stuttering [20, 30]. To ensure objectivity and the reproducibility of these measurements, all clinical interviews were audio‐recorded. The %SS values initially evaluated by the first author on the day of the consultation were subsequently reviewed and verified by two co‐authors, both of whom are certified speech‐language‐hearing therapists to reach a consensus.

#### 2.3.2 Extraction and Classification of Reasonable Accommodations (Content Analysis)

Inductive content analysis was performed to systematize the reasonable accommodations described in the letters [31].

Objectivity and reliability were ensured by having two speech‐language‐hearing therapists, who were not involved in drafting the letters, independently extract text describing reasonable accommodations. Inter‐rater reliability for the extraction process was high, with a Cohen's kappa coefficient of 0.83. The extracted text data were analyzed using the following steps:

- Open coding involved extracting key meaning units (codes) from the descriptions.
- Categorization involved aggregating similar codes into subcategories, which were then abstracted into main categories.
- Validation involved repeated discussions among researchers with expertise in stuttering clinical practice and research regarding the classification process and the naming of codes, subcategories, and categories. Reliability was ensured by refining criteria through team discussions until a consensus was reached.

### 2.4 Statistical Analysis

Differences in the proportions of accommodation requests between the elementary and secondary school groups were analyzed using Fisher's exact tests (or chi‐square tests where appropriate). Stuttering frequency (%SS) [20, 30] and social anxiety (LSAS‐CA) scores [20, 32] were summarized as descriptive statistics (means, standard deviations [SD], and ranges) to characterize the clinical profile of the participants. Statistical significance was defined as *p* < 0.05. All analyses were performed using JMP Pro version 17 (SAS Institute Inc., Cary, NC, USA).

### 2.5 Ethical Considerations

This study was conducted in accordance with the Declaration of Helsinki and was approved by the Kyushu University Institutional Review Board (Approval no. 2021‐5). All personal information was anonymized to protect participants' privacy.

### 2.6 Informed Consent

An opt‐out method was used to obtain informed consent due to the retrospective nature of the study. Information regarding the study was publicized on our institutional website to provide participants with the opportunity to decline. Consent for publication was also obtained from all participants via this opt‐out procedure.

## 3 Results

### 3.1 Clinical Characteristics of Participants

Regarding the clinical characteristics of the participants, the mean stuttering frequency (%SS) was 5.2% (standard deviation [SD] = 4.1%, range: 0.5%–21.3%). For the older students who completed the social anxiety assessment (*n* = 69), the mean Liebowitz Social Anxiety Scale for Children and Adolescents (LSAS‐CA) score was 42.1 (SD = 21.8, range: 8–98).

### 3.2 Content of Reasonable Accommodations

Content analysis of 116 letters identified five categories and 21 subcategories of reasonable accommodations. Table 1 presents the percentage (counts) of accommodation requests in the elementary school group (*n* = 65) and the secondary school group (*n* = 51). Overall, the request rates for the five main categories were 56.9% (*n* = 66) for teasing/bullying and classroom explanation, 61.2% (*n* = 71) for accommodations for presentations and class activities, 44.8% (*n* = 52) for sharing information with teachers other than the homeroom teacher, 12.1% (*n* = 14) for extracurricular activities and school events, and 9.5% (*n* = 11) for out‐of‐school examinations and entrance exams.

**TABLE 1:** Number of accommodations by school level in the five categories and 21 subcategories.

Category/sub‐category	Elementary (*n* = 65)	Secondary (*n* = 51)
*Reporting teasing/bullying to the teacher*		
Reporting being pointed out about way of speaking	50.8% (33)	13.7% (7)
Reporting being imitated	40.0% (26)	9.8% (5)
Reporting being laughed at	32.3% (21)	11.8% (6)
Reporting being told nasty words	12.3% (8)	7.8% (4)
Reporting unpleasant physical contact	3.1% (2)	2.0% (1)
*Teacher explains stuttering*		
Teacher explains stuttering to the class	43.1% (28)	5.9% (3)
Teacher explains stuttering to students individually	12.3% (8)	3.9% (2)
*Accommodations for presentations*		
Accommodations for presentations (other than reading aloud)	32.3% (21)	29.4% (15)
Accommodations for reading textbooks aloud	23.1% (15)	19.6% (10)
Accommodations for timed presentations	15.4% (10)	5.9% (3)
Accommodations for day duty (e.g., chairing, commands)	13.8% (9)	13.7% (7)
Accommodations for reply to roll call (health observation)	6.2% (4)	0.0% (0)
Accommodations for presentations at special events	4.6% (3)	7.8% (4)
Exemption from presentation	4.6% (3)	5.9% (3)
Accommodations for group work	0.0% (0)	17.6% (9)
Accommodations for self‐introductions	0.0% (0)	13.7% (7)
*Sharing information with teachers other than the homeroom teacher*		
Sharing information with subject teachers	18.5% (12)	35.3% (18)
Sharing information with teachers near the staff room entrance	0.0% (0)	13.7% (7)
Sharing information with club activity teachers	0.0% (0)	9.8% (5)
*Accommodations for interviews/exams outside the enrolled school*		
Accommodations for English proficiency tests	0.0% (0)	19.6% (10)
Accommodations for entrance examination interviews	0.0% (0)	13.7% (7)

*Note:* Values indicate percentage (number of students). Secondary: Includes junior high and high school students. Percentages for main categories represent the proportion of students requesting at least one accommodation within that category. Percentages do not sum to 100% because individual students could request multiple accommodations across subcategories.

#### 3.2.1 Teasing/Bullying and Classroom Explanation

In the category “Reporting teasing or bullying to the teacher,” the proportion of requests was higher in the elementary school group than in the secondary school group across all subcategories. Notably, requests to report being singled out for manner of speaking were most frequent in the elementary school group (50.8%), representing a marked difference compared with the secondary school group (13.7%). Similarly, within the category “Teacher explains stuttering,” requests for teachers to explain stuttering to the class were reported in 43.1% of the elementary school group but only 5.9% of the secondary school group.

#### 3.2.2 Accommodations for Presentations and Class Activities

Requests for accommodations during presentations (e.g., exemption from reading aloud or alternative formats) were common in both groups (Elementary: 32.3%, Secondary: 29.4%). However, the specific contexts differed. Requests related to “Group work” (17.6%) and “Self‐introductions” (13.7%) were observed exclusively in the secondary school group. Conversely, accommodations for “Timed presentations” (e.g., reading within a time limit) were more frequent in the elementary school group (15.4%) than in the secondary school group (5.9%). Requests regarding “Day duty” (Nichoku) tasks, such as leading class greetings, were similar between groups (approx. 14%).

#### 3.2.3 Information Sharing and Extracurricular Activities

Within the category “Sharing information with teachers other than the homeroom teacher,” requests involving subject teachers accounted for 35.3% of the secondary school group, nearly double the 18.5% observed in the elementary school group. Requests involving teachers near the staff room entrance and club activity teachers were reported exclusively in the secondary school group. Additionally, requests for accommodations related to interviews or examinations outside the enrolled school, including English proficiency tests and entrance examinations, were observed exclusively in the secondary school group.

## 4 Discussion

This study examined the characteristics of reasonable accommodations requested by elementary and secondary school students who stutter in Japan through a content analysis of Medical Letters. Unlike previous studies that relied on student self‐reports [26], this study provides a unique perspective by analyzing accommodations formulated through “shared decision‐making” between a specialist physician, students, and their guardians. The findings indicate that accommodation needs are multifaceted—influenced significantly by the school system's structure and developmental stage, alongside the students' clinical and psychological profiles.

### 4.1 Developmental Differences and School System Structure

Distinct patterns of requested accommodations were observed between elementary and secondary school students, reflecting the structural transition in the Japanese education system. Elementary school students more frequently requested teacher‐mediated support, such as reporting teasing and explaining stuttering to the class. In the Japanese elementary “homeroom system,” the class teacher plays a central, protective role in peer dynamics. Therefore, requests for the teacher to explain stuttering likely represent strategic efforts to utilize the teacher's authority to educate peers and prevent bullying [25, 27]. In contrast, secondary school students significantly more frequently requested information sharing with subject teachers. This shift corresponds to the “subject teacher system” in secondary education, where students interact with multiple teachers daily. As the protective reach of a single homeroom teacher diminishes, systematic information sharing becomes essential to ensure consistent support across different classes. Furthermore, the emergence of requests regarding entrance examinations reflects the high‐stakes nature of academic progression in Japan, where interview performance can determine future educational paths.

These findings align with and extend previous survey‐based research on support requests among school‐age children who stutter in Japan. For instance, Iimura et al. (2021) identified widespread needs for presentation accommodations and teacher‐mediated support through a free‐text questionnaire completed by students and parents [26]. While their survey captured subjective, self‐reported desires directly from families, our study analyzed formal medical letters formulated through clinician‐guided shared decision‐making. Despite these methodological differences, both studies consistently demonstrate that presentation accommodations and classroom explanations represent primary areas of concern. Crucially, our study extends prior literature by demonstrating how these requested accommodations shift systematically across developmental stages—from direct, teacher‐mediated protection under the elementary homeroom system to broader, subject‐teacher information sharing in secondary school. This suggests that formal medical letters reflect not only individual subjective preferences but also medically and educationally validated adjustments tailored to the student's changing educational environment.

### 4.2 Social Anxiety and Presentation‐Related Accommodations

One notable observation was that requests for presentation‐related accommodations were highly prevalent (overall, 61.2%), despite the structural differences in school environments. Although we did not perform a direct association analysis, our descriptive data showed that students who completed the anxiety assessment exhibited elevated social anxiety, with a mean LSAS‐CA score of 42.1. This high rate of presentation‐related requests likely reflects the substantial subjective burden and functional impairment experienced by students who stutter during public speaking, which does not or may not align with observable speech symptoms. Students who appear fluent in clinical settings may nonetheless experience substantial anxiety in school‐based speaking situations due to covert coping strategies, such as word substitution or excessive filler use. Given evidence that SAD is common among adolescents who stutter and is associated with adverse educational outcomes, including school refusal [20], clinicians and educators should prioritize presentation accommodations based on the student's internal anxiety and subjective distress rather than relying solely on objective stuttering frequency.

### 4.3 Medical Letters as a Tool Within the Social Model of Disability

Since the enforcement of the Act for Eliminating Discrimination against Persons with Disabilities in Japan, the provision of reasonable accommodations has expanded; however, practical barriers persist. The medical letters analyzed in this study should be viewed not merely as diagnostic certificates but as communication tools that translate clinical knowledge into concrete, context‐specific recommendations for educational settings. In this sense, these letters may serve as a bridge between the medical and social models of disability by shifting attention from individual impairment to modifiable environmental barriers. However, this study did not assess how these letters were interpreted or implemented by schools, and their actual impact remains unknown.

### 4.4 Medical Letters as Tools for “Constructive Dialogue”

A critical implication of this study is the redefined role of medical letters. Rather than being unilateral “orders” from a physician, these letters serve as tools to initiate “constructive dialogue” between the school and the student. Since the enforcement of the Act for Eliminating Discrimination against Persons with Disabilities, schools are legally obligated to provide accommodations. However, teachers often lack specific knowledge of stuttering, and students may lack the self‐advocacy skills to articulate their needs. In this context, medical letters drafted by specialists act as a “needs assessment” that translates clinical insights into concrete educational requests. By explicitly documenting these needs, the letters bridge the gap between the medical model (diagnosis) and the social model (environmental adjustment), empowering students to participate fully in school life [28].

## 5 Limitations

Several limitations warrant consideration. First, although the single‐physician design ensured consistency in clinical assessment criteria, it may also introduce bias related to the specific counseling style or “prescribing habits” of the drafter. However, this risk was mitigated by the participatory drafting process, where students and guardians reviewed and revised the draft, ensuring the content reflected their subjective needs rather than solely the physician's perspective. Future studies should involve multiple centers to verify generalizability. Second, this study focused on “requested” accommodations (needs assessment) rather than “implemented” accommodations (outcomes). Whether these requests were accepted by schools and whether they successfully reduced student distress remains a subject for future investigation. Third, the LSAS‐CA data were limited to a sub‐sample of older students, preventing a full analysis of how social anxiety interacts with accommodation needs in younger children. Fourth, this study analyzed only students who stutter for whom a medical letter was drafted and did not include a comparison group of students who stuttered but did not request or require a medical letter. Consequently, our findings reflect the specific needs of a clinical population seeking formal accommodations rather than the general population of students who stutter.

## 6 Conclusions

Accommodation needs for students who stutter are dynamic and context‐dependent. They shift from protection and peer education in the elementary homeroom environment to independence and information sharing in the secondary subject‐teacher system. Importantly, presentation‐related accommodations represent a highly prevalent and consistent area of need across all school levels. Clinicians and educators must recognize these developmental and structural factors to provide effective, individualized support.

## Author Contributions

Y.K., Y.Y. designed the study. Y.K., Y.Y., A.S. performed the experiments and collected and analyzed the data. Y.K. wrote the manuscript. M.K., E.Y., T.N. provided conceptual advice. All authors read and approved the final manuscript.

## Funding

This work was supported by Japan Society for the Promotion of Science (JP24K00440).

## Conflicts of Interest

The authors declare no conflicts of interest.

## Data Availability

The data that support the findings of this study are available from the corresponding author upon reasonable request.
